# Supplementary material for: The Evolution and Characterization of the RNA Interference Pathways in Lophotrochozoa
Source: Genome Biol Evol. 2024 May 7;16(5):evae098. doi: 10.1093/gbe/evae098 (PMC11114477; doi:10.1093/gbe/evae098)
Supplement: evae098_Supplementary_Data [file evae098_supplementary_data.zip › Supplementary_materials.zip!/selected_miRNAs/NoGe_selected_miRNAs/mirdeep_pdf_files/NMRB01000117.1_Notospermus_geniculatus_strain_Ushimado_scaffold117,_whole_genome_shotgun_sequence_4718.pdf]

| | | |
|---|---|---|
| Provisional ID | : | NMRB01000117.1_Notospermus_geniculatus_strain_Ushimado_scaffold117,_whole_genome_shotgun_sequence_4718 |
| Score total | : | 25499.6 |
| Score for star read(s) | : | 3.9 |
| Score for read counts | : | 25491.4 |
| Score for mfe | : | 1.3 |
| Score for randfold | : | 0 |
| Score for cons. seed | : | 3 |
| Total read count | : | 50012 |
| Mature read count | : | 49980 |
| Loop read count | : | 0 |
| Star read count | : | 32 |

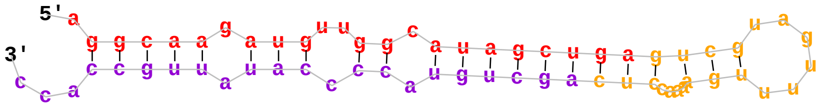


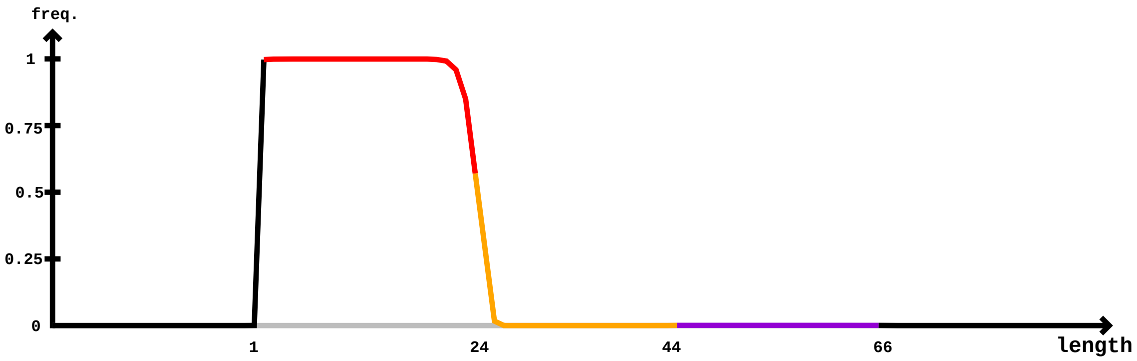


```
                         Mature                                Star
5'- ucgagacaugaacucagauuaggcaagauguuggcauagcugagucguaguuuugaaaaccucagcuguaccccauauugccaccuuuggguuuauaaaauucaacaug  -3'   obs
    ucgagacaugaacucagauuaggcaagauguuggcauagcugagucguaguuuugaaaaccucagcuguaccccauauugccaccuuuggguuuauaaaauucaacaug        exp
    ..(((..((((((((((...(((((.(((..((.((((((((((((......)))....))))))))).)).))).)))))...))))))))))...))).....   reads    mm    sample
    ...................Caggcaagauguuggcauagcuga..................................................................   2        1     Fm1
    ....................aggcaagauguuggcaua.......................................................................   5        0     Fm1
    ....................aggcaagauguuggcauag......................................................................   9        0     Fm1
    ....................aggcaagauguuggcauagc.....................................................................   66       0     Fm1
    ....................aggcaagauguuggcauagU.....................................................................   13       1     Fm1
    ....................aggcaagauguuggcauagcu....................................................................   329      0     Fm1
    ....................aggcaagauguuggcauagUu....................................................................   2        1     Fm1
    ....................Nggcaagauguuggcauagcu....................................................................   1        1     Fm1
    ....................aggcaagauguuggcaAagcu....................................................................   1        1     Fm1
    ....................Uggcaagauguuggcauagcu....................................................................   1        1     Fm1
    ....................aggcaagauguugAcauagcu....................................................................   1        1     Fm1
    ....................aggcaagauguuggcauagcG....................................................................   27       1     Fm1
    ....................aggcaagauguuggcauagcA....................................................................   1        1     Fm1
    ....................aggcaagauguuggcauagcuC...................................................................   7        1     Fm1
    ....................aggcaagauguuggcauagAug...................................................................   2        1     Fm1
    ....................aggcaagauguuggcaCagcug...................................................................   10       1     Fm1
    ....................aggcaagauguuggcauagcGg...................................................................   48       1     Fm1
    ....................aggcaagauguuggcauagcuA...................................................................   8        1     Fm1
    ....................aggcaUgauguuggcauagcug...................................................................   2        1     Fm1
    ....................aggcaagaugAuggcauagcug...................................................................   6        1     Fm1
    ....................Uggcaagauguuggcauagcug...................................................................   1        1     Fm1
    ....................agCcaagauguuggcauagcug...................................................................   1        1     Fm1
    ....................aggcaagauguuggcauCgcug...................................................................   11       1     Fm1
    ....................aggcaagauguuggcauagUug...................................................................   5        1     Fm1
    ....................aggcaagauguuggcaGagcug...................................................................   8        1     Fm1
    ....................aggcaagauUuuggcauagcug...................................................................   1        1     Fm1
    ....................aggcaagauguuggcauagcug...................................................................   2594     0     Fm1
    ....................aggcaagaugCuggcauagcug...................................................................   5        1     Fm1
    ....................aggcaagauguuggcauGgcug...................................................................   7        1     Fm1
    ....................aggcaagauguuUgcauagcug...................................................................   1        1     Fm1
    ....................aggcaagUuguuggcauagcug...................................................................   2        1     Fm1
    ....................aggcaagauguuggcauagcuU...................................................................   42       1     Fm1
    ....................aggcaagauguuggAauagcug...................................................................   2        1     Fm1
```

```
                    Mature                                    Star
ucgagacaugaacucagauuaggcaagauguuggcauagcugagucguaguuuugaaaaccucagcuguaccccauauugccaccuuuggguuuauaaauucaacaug
....................aggcCagauguuggcauagcug..................................................................  1     1   Fm1
....................Nggcaagauguuggcauagcug..................................................................  1     1   Fm1
....................aggcaagauguuggUauagcug..................................................................  11    1   Fm1
....................aUgcaagauguuggcauagcug..................................................................  3     1   Fm1
....................aggcaagauguuggcaAagcug..................................................................  3     1   Fm1
....................aggcaNgauguuggcauagcug..................................................................  1     1   Fm1
....................aggcaGgauguuggcauagcug..................................................................  2     1   Fm1
....................aggcaagauguuggcauagGug..................................................................  1     1   Fm1
....................aggcaagauguuAgcauagcug..................................................................  1     1   Fm1
....................aggcaagaugGuggcauagcug..................................................................  1     1   Fm1
....................aggcaagauguuggcauUgcug..................................................................  4     1   Fm1
....................aggcaagaugCuggcauagcuga.................................................................  3     1   Fm1
....................aggcaagauguuggcauagcugC.................................................................  4     1   Fm1
....................aggcaagauguuggcauCgcuga.................................................................  5     1   Fm1
....................aggcaagauguuUgcauagcuga.................................................................  1     1   Fm1
....................aggcaagauguuggcauaUcuga.................................................................  2     1   Fm1
....................agCcaagauguuggcauagcuga.................................................................  1     1   Fm1
....................aggcaagCuguuggcauagcuga.................................................................  1     1   Fm1
....................aggcaagauguuggcauagUuga.................................................................  1     1   Fm1
....................aggcaagauguuggcaGagcuga.................................................................  7     1   Fm1
....................aggcaagauguuggcauagcuga.................................................................  2039  0   Fm1
....................aggcaagauguuggcauagcugG.................................................................  88    1   Fm1
....................aggcaagauguuggcaCagcuga.................................................................  4     1   Fm1
....................aggcaagauguuggcaAagcuga.................................................................  7     1   Fm1
....................aggcaagauguuggcauGgcuga.................................................................  8     1   Fm1
....................aggcaagauguuggUauagcuga.................................................................  1     1   Fm1
....................aggcCagauguuggcauagcuga.................................................................  1     1   Fm1
....................aggcaagauguuAgcauagcuga.................................................................  1     1   Fm1
....................aggcaagauguuggcauagcuUa.................................................................  3     1   Fm1
....................aggcaagauguuggcauagcuCa.................................................................  1     1   Fm1
....................aggcaagauguuggcauagcugU.................................................................  746   1   Fm1
....................aggcaUgauguuggcauagcuga.................................................................  1     1   Fm1
....................Uggcaagauguuggcauagcuga.................................................................  4     1   Fm1
....................Gggcaagauguuggcauagcuga.................................................................  1     1   Fm1
....................aggcaagauguuggcauagcuAa.................................................................  6     1   Fm1
....................aggcaagauguuggcauUgcuga.................................................................  3     1   Fm1
....................aggcaagaugGuggcauagcuga.................................................................  5     1   Fm1
....................aggcaagaugAuggcauagcuga.................................................................  2     1   Fm1
....................agAcaagauguuggcauagcuga.................................................................  2     1   Fm1
....................aggcaagauguuggcUuagcuga.................................................................  2     1   Fm1
....................aggcaaNauguuggcauagcuga.................................................................  1     1   Fm1
....................agUcaagauguuggcauagcuga.................................................................  1     1   Fm1
....................aggcaagauguuggcauagcugaC................................................................  3     1   Fm1
....................aggcaagauguuggcauagcugaU................................................................  294   1   Fm1
....................aggcaagauguuggcauagcugaA................................................................  7     1   Fm1
....................aggcaagauguuggcauagcugaUu...............................................................  93    1   Fm1
....................aggcaagauguuggcauagcugaAu...............................................................  22    1   Fm1
....................aggcaagauguuggcauagcugagu...............................................................  4     0   Fm1
....................Gggcaagauguuggcauagcugagu...............................................................  1     1   Fm1
.....................ggcaagauguuggcauagcuga.................................................................  3     0   Fm1
......................gcaagauguuggcauagcugaU................................................................  4     1   Fm1
...............................................................agcuguaccccauauugAcacc.......................  2     1   Fm1
...............................................................agcuguaccccauauugccacc.......................  9     0   Fm1

....................aggcaagauguuggcaua......................................................................  5     0   Fm2
....................aggcaagauguuggcauag.....................................................................  17    0   Fm2
....................aggcaagauguuggcauagU....................................................................  11    1   Fm2
....................aggcaagauguuggcauagc....................................................................  125   0   Fm2
....................aggcaagauguuggcauCgc....................................................................  1     1   Fm2
....................aggcaagauguuggcaCagc....................................................................  1     1   Fm2
....................aggcaagauguuggcaCagcu...................................................................  1     1   Fm2
....................aggcaagauguuggcauagcC...................................................................  1     1   Fm2
....................aggcaagaugCuggcauagcu...................................................................  1     1   Fm2
....................aggcaagauguuggcauagcu...................................................................  294   0   Fm2
....................aggcaagauguuggcauUgcu...................................................................  1     1   Fm2
....................aggcaCgauguuggcauagcu...................................................................  1     1   Fm2
....................aggcaagaAguuggcauagcu...................................................................  1     1   Fm2
....................aggcGagauguuggcauagcu...................................................................  1     1   Fm2
....................aggcaagaugAuggcauagcu...................................................................  1     1   Fm2
....................aggcaagauguuUgcauagcu...................................................................  2     1   Fm2
```

Mature                                    Star

```
ucgagacaugaacucagauuaggcaagauguuggcauagcugagucguaguuuugaaaaccucagcuguaccccauauugccaccuuuggguuuauaaauucaacaug
....................aggcaagauguuggcauGgcu...................................................................   1      1     Fm2
....................aggcaagauguuggcauagcG...................................................................   29     1     Fm2
....................aggcaagauguuggcauagUu...................................................................   1      1     Fm2
....................aggcaagaugGuggcauagcu...................................................................   1      1     Fm2
....................aggcaagauguuggcaAagcu...................................................................   2      1     Fm2
....................aggcaagauguuggcauCgcu...................................................................   1      1     Fm2
....................aggcaagauguuggcauCgcug..................................................................   10     1     Fm2
....................aggcaagauguuggcaCagcug..................................................................   7      1     Fm2
....................aggcaagaugCuggcauagcug..................................................................   1      1     Fm2
....................aggcaagauguuggcaAagcug..................................................................   2      1     Fm2
....................aggcaagauguuggcauagcGg..................................................................   56     1     Fm2
....................aggcaagauguuggcauagcuA..................................................................   2      1     Fm2
....................agAcaagauguuggcauagcug..................................................................   1      1     Fm2
....................aggcaagauguCggcauagcug..................................................................   1      1     Fm2
....................aggcGagauguuggcauagcug..................................................................   1      1     Fm2
....................aggcaagauguAggcauagcug..................................................................   1      1     Fm2
....................aggcaagauguuUgcauagcug..................................................................   1      1     Fm2
....................aggcaagauguuggcGuagcug..................................................................   1      1     Fm2
....................aggcaagauguuggcaGagcug..................................................................   7      1     Fm2
....................aggcaagaugAuggcauagcug..................................................................   2      1     Fm2
....................aggcaagaugGuggcauagcug..................................................................   1      1     Fm2
....................aggcaUgauguuggcauagcug..................................................................   1      1     Fm2
....................agUcaagauguuggcauagcug..................................................................   2      1     Fm2
....................Gggcaagauguuggcauagcug..................................................................   1      1     Fm2
....................aggcaagauguuggcauagcug..................................................................   2520   0     Fm2
....................aAgcaagauguuggcauagcug..................................................................   3      1     Fm2
....................aggcaaAauguuggcauagcug..................................................................   1      1     Fm2
....................aggcaagaGguuggcauagcug..................................................................   1      1     Fm2
....................aggcaagauguuggcauagcuU..................................................................   62     1     Fm2
....................aggcaagauguuggUauagcug..................................................................   1      1     Fm2
....................aggcaagauguuggcauagcCg..................................................................   3      1     Fm2
....................aggcaagauguuggcauGgcug..................................................................   10     1     Fm2
....................aggcaagauguuggcauUgcug..................................................................   5      1     Fm2
....................aggcaagauguuggcauagUuga.................................................................   2      1     Fm2
....................aggcaaNauguuggcauagcuga.................................................................   1      1     Fm2
....................aggcaagauguuggcaGagcuga.................................................................   3      1     Fm2
....................aggcaagauguuggcauagcugG.................................................................   91     1     Fm2
....................aggcaaUauguuggcauagcuga.................................................................   1      1     Fm2
....................agAcaagauguuggcauagcuga.................................................................   1      1     Fm2
....................aggcaagauguuggcauUgcuga.................................................................   4      1     Fm2
....................aggcaagauguuggcauaUcuga.................................................................   1      1     Fm2
....................aggcaagaugGuggcauagcuga.................................................................   5      1     Fm2
....................aggcaagauguuggcauaAcuga.................................................................   1      1     Fm2
....................aggcaagauguuggcauagcCga.................................................................   3      1     Fm2
....................aggcaaAauguuggcauagcuga.................................................................   1      1     Fm2
....................aggcaagauguuggcGuagcuga.................................................................   2      1     Fm2
....................Nggcaagauguuggcauagcuga.................................................................   1      1     Fm2
....................aggcaagauguuggcaCagcuga.................................................................   6      1     Fm2
....................aggcaagauguuggcauGgcuga.................................................................   9      1     Fm2
....................aggcaagCuguuggcauagcuga.................................................................   1      1     Fm2
....................aggcaagauguuggcauagcuga.................................................................   2826   0     Fm2
....................aggcaagauguuggcauCgcuga.................................................................   9      1     Fm2
....................Uggcaagauguuggcauagcuga.................................................................   1      1     Fm2
....................aggcaagauguuggcauagcuUa.................................................................   2      1     Fm2
....................aUgcaagauguuggcauagcuga.................................................................   1      1     Fm2
....................aggcaagGuguuggcauagcuga.................................................................   1      1     Fm2
....................aggcaagauguuUgcauagcuga.................................................................   2      1     Fm2
....................aggcaaCauguuggcauagcuga.................................................................   1      1     Fm2
....................aggcaagauguuCgcauagcuga.................................................................   1      1     Fm2
....................aggcaNgauguuggcauagcuga.................................................................   1      1     Fm2
....................aggcaagaugCuggcauagcuga.................................................................   4      1     Fm2
....................aggcaagauguuggcauagcugU.................................................................   436    1     Fm2
....................aggcaagaugAuggcauagcuga.................................................................   10     1     Fm2
....................aggcaagauguuggcauagcugaU................................................................   188    1     Fm2
....................aggcaagauguuggcauagcugaUu...............................................................   77     1     Fm2
.....................ggcaagauguuggcauagcug..................................................................   13     0     Fm2
.....................ggcaagauguuggcauagcuga.................................................................   3      0     Fm2
......................gcaagauguuggcauagcuga.................................................................   8      0     Fm2

...................uaggcaagauguuggcauagcuga.................................................................   1      0     Mm2
```

```
                    Mature                                    Star
ucgagacaugaacucagauuaggcaagauguuggcauagcugagucguaguuuugaaaaccucagcuguaccccauauugccaccuuggguuuauaaauucaacaug
....................aggcaagauguuggcauU.....................................................................   1      1      Mm2
....................aggcaagauguuggcaua.....................................................................   1      0      Mm2
....................aggcaagauguuggcauag....................................................................   11     0      Mm2
....................aggcaagauguuggcauagA...................................................................   1      1      Mm2
....................aggcaagaugGuggcauagc...................................................................   1      1      Mm2
....................aggcaagauguuggcaAagc...................................................................   1      1      Mm2
....................aggcaagauguuggcauagc...................................................................   49     0      Mm2
....................aggcaagauguuggcauagU...................................................................   11     1      Mm2
....................aggcaagauguuggcauagcA..................................................................   1      1      Mm2
....................aggcaUgauguuggcauagcu..................................................................   1      1      Mm2
....................aggcaagauguuggcauagGu..................................................................   1      1      Mm2
....................aggcaagauguuggcauagcu..................................................................   203    0      Mm2
....................aggcaagauguuggcauagcG..................................................................   14     1      Mm2
....................aggcaagauguuggcauGgcu..................................................................   1      1      Mm2
....................aggcaagauguuggcauagUu..................................................................   3      1      Mm2
....................aggcaagauguuggcauagcCg.................................................................   1      1      Mm2
....................aggcaagauguuggcauUgcug.................................................................   3      1      Mm2
....................aggcaagauguuggcauGgcug.................................................................   2      1      Mm2
....................aggcaagaugGuggcauagcug.................................................................   1      1      Mm2
....................aggcaagauguuggcauagcGg.................................................................   8      1      Mm2
....................aggcaagaugAuggcauagcug.................................................................   2      1      Mm2
....................aggcaagauguuggcauagcug.................................................................   741    0      Mm2
....................aggcaagauguuggcauagcuU.................................................................   31     1      Mm2
....................aggcaagauguuggcauagcAg.................................................................   1      1      Mm2
....................aggcaagauguuggcauCgcug.................................................................   4      1      Mm2
....................aggcaaUauguuggcauagcug.................................................................   1      1      Mm2
....................aggcaaNauguuggcauagcug.................................................................   1      1      Mm2
....................aggcaagauguuggcaGagcug.................................................................   2      1      Mm2
....................aggcaagauguuggcauagcuC.................................................................   1      1      Mm2
....................aggcaagauguuggcauagcuA.................................................................   3      1      Mm2
....................Gggcaagauguuggcauagcug.................................................................   1      1      Mm2
....................aggcaagauguuggcaAagcug.................................................................   1      1      Mm2
....................aggcaagauguuggcaAagcuga................................................................   4      1      Mm2
....................aggcaagauguuggcaGagcuga................................................................   5      1      Mm2
....................aggcaagaugAuggcauagcuga................................................................   3      1      Mm2
....................agAcaagauguuggcauagcuga................................................................   1      1      Mm2
....................Cggcaagauguuggcauagcuga................................................................   1      1      Mm2
....................aggcaagauguuggcauGgcuga................................................................   6      1      Mm2
....................aggcaagauguuggcauagcuga................................................................   1168   0      Mm2
....................aggcaagauguuggcauagcuUa................................................................   2      1      Mm2
....................aggcaGgauguuggcauagcuga................................................................   1      1      Mm2
....................aggcaagauguuggcauUgcuga................................................................   2      1      Mm2
....................aggcaagUuguuggcauagcuga................................................................   1      1      Mm2
....................aggcaagauguuggcauagcugC................................................................   1      1      Mm2
....................aggAaagauguuggcauagcuga................................................................   1      1      Mm2
....................Uggcaagauguuggcauagcuga................................................................   1      1      Mm2
....................aggcaagauguuggcauagcugG................................................................   33     1      Mm2
....................aggcaagaugGuggcauagcuga................................................................   1      1      Mm2
....................aggcaagauguuggcaCagcuga................................................................   1      1      Mm2
....................aggcaagauguugAcauagcuga................................................................   1      1      Mm2
....................aggcaagauguuUgcauagcuga................................................................   1      1      Mm2
....................aggcaagaugCuggcauagcuga................................................................   1      1      Mm2
....................aggcaagauguuggAauagcuga................................................................   1      1      Mm2
....................aggcaagauguuggcGuagcuga................................................................   1      1      Mm2
....................aggcaagauguuggcauCgcuga................................................................   3      1      Mm2
....................aggcaagauguuggcauagcugU................................................................   344    1      Mm2
....................aggcaagauguuggcauagcugaA...............................................................   3      1      Mm2
....................aggcaagauguuggcauagcugaU...............................................................   80     1      Mm2
....................aggcaagauguuggcauagcugaUu..............................................................   33     1      Mm2
....................aggcaagauguuggcauagcugaAu..............................................................   11     1      Mm2
....................aggcaagauguuggcauagcugagu..............................................................   2      0      Mm2
.....................ggcaagauguuggcauagcugU................................................................   1      1      Mm2
.....................ggcaagauguuggcauagcuga................................................................   4      0      Mm2
.......................caagauguuggcauagcugagC..............................................................   1      1      Mm2
...............................................................agcuguaccccauauugccacc......................   5      0      Mm2
...............................................................Ugcuguaccccauauugccacc......................   1      1      Mm2
...............................................................agcuguaccccauauugccaccA.....................   1      1      Mm2

....................aggcaagauguuggcauagU...................................................................   2      1      Mm1
....................aggcaagauguuggcauagcu..................................................................   23     0      Mm1
```

```
                    Mature                                    Star
ucgagacaugaacucagauuaggcaagauguuggcauagcugagucguaguuuugaaaaccucagcuguaccccauauugccaccuuuggguuuauaaaauucaacaug
....................aggcaagauguuggcauagcG....................................................................   1     1    Mm1
....................aggcaagauguuggcauagcug...................................................................   227   0    Mm1
....................aggcaagauguuggcauCgcug...................................................................   1     1    Mm1
....................Gggcaagauguuggcauagcug...................................................................   1     1    Mm1
....................aggcaagauguuggcauagcuU...................................................................   21    1    Mm1
....................aggcaagauguuggcauagcGg...................................................................   1     1    Mm1
....................aggcaagauguuggcaGagcug...................................................................   1     1    Mm1
....................aggcaagauguuggcaCagcug...................................................................   3     1    Mm1
....................aggcaagauguuggcauUgcug...................................................................   1     1    Mm1
....................aggcaagaugGuggcauagcuga..................................................................   1     1    Mm1
....................aggcaagauguuggcaAagcuga..................................................................   2     1    Mm1
....................aggcaagauguuggcauagcugU..................................................................   106   1    Mm1
....................aggcaagauguuggcauagcuga..................................................................   251   0    Mm1
....................Uggcaagauguuggcauagcuga..................................................................   1     1    Mm1
....................aggcaagauguuggcauGgcuga..................................................................   1     1    Mm1
....................aggcaagauguuggcauagcugG..................................................................   1     1    Mm1
....................aggcaagauguuggcauagcugaU.................................................................   5     1    Mm1
....................aggcaagauguuggcauagcugagu................................................................   4     0    Mm1
....................aggcaagauguuggcauagcugaUu................................................................   18    1    Mm1

...................uaggcaagauguuggcauagcu....................................................................   5     0    Fm3
...................Caggcaagauguuggcauagcug...................................................................   2     1    Fm3
...................uaggcaagauguuggcauagcug...................................................................   3     0    Fm3
....................aggcaagauguuggcaGa.......................................................................   1     1    Fm3
....................aggcaagauguuggcauC.......................................................................   1     1    Fm3
....................aggcaagauguuggcauU.......................................................................   16    1    Fm3
....................aggcaagauguuggcaua.......................................................................   55    0    Fm3
....................aggcaagauguuggcaAag......................................................................   2     1    Fm3
....................aggcaagauguuggcGuag......................................................................   1     1    Fm3
....................aggcaagaugAuggcauag......................................................................   4     1    Fm3
....................aggcaagauguuggcauag......................................................................   233   0    Fm3
....................aAgcaagauguuggcauag......................................................................   1     1    Fm3
....................aggcaagauguuggcauagU.....................................................................   174   1    Fm3
....................aggcaagauguuggcaCagc.....................................................................   2     1    Fm3
....................Uggcaagauguuggcauagc.....................................................................   1     1    Fm3
....................aggcaagauguuggcauUgc.....................................................................   1     1    Fm3
....................aAgcaagauguuggcauagc.....................................................................   2     1    Fm3
....................aggcaagauguuggcauagc.....................................................................   1074  0    Fm3
....................aggcaagauguuCgcauagc.....................................................................   1     1    Fm3
....................aggcaagauguuggcaGagc.....................................................................   4     1    Fm3
....................aUgcaagauguuggcauagc.....................................................................   1     1    Fm3
....................aggcaagauguuggcauCgc.....................................................................   1     1    Fm3
....................aggcaagaugAuggcauagc.....................................................................   2     1    Fm3
....................aggcGagauguuggcauagc.....................................................................   1     1    Fm3
....................aggcaagauguuggcauGgc.....................................................................   5     1    Fm3
....................aggcaagauguuggcauagA.....................................................................   6     1    Fm3
....................aggcaagauguuggUauagc.....................................................................   3     1    Fm3
....................aggcaagauguuggcaAagc.....................................................................   3     1    Fm3
....................aggcaagauguuggcauaCcu....................................................................   1     1    Fm3
....................aggcaagauguuggcauaUcu....................................................................   2     1    Fm3
....................aggcaaNauguuggcauagcu....................................................................   1     1    Fm3
....................aggcaagaugCuggcauagcu....................................................................   2     1    Fm3
....................aggcCagauguuggcauagcu....................................................................   1     1    Fm3
....................aggcaagauguuUgcauagcu....................................................................   4     1    Fm3
....................aggcaagaugGuggcauagcu....................................................................   3     1    Fm3
....................aggcaagauguCggcauagcu....................................................................   1     1    Fm3
....................aggcaagauguuggcauagcA....................................................................   8     1    Fm3
....................aggcaagauguuggcauagUu....................................................................   28    1    Fm3
....................aggcaagauAuggcauagcu.....................................................................   2     1    Fm3
....................aggcaagauguuggcaCagcu....................................................................   17    1    Fm3
....................aggcaagauguGggcauagcu....................................................................   1     1    Fm3
....................aggcaaUauguuggcauagcu....................................................................   1     1    Fm3
....................aggcaagauguuggcauUgcu....................................................................   14    1    Fm3
....................aAgcaagauguuggcauagcu....................................................................   2     1    Fm3
....................aggcaagauguuggcaAagcu....................................................................   9     1    Fm3
....................aggcaagauguugAcauagcu....................................................................   1     1    Fm3
....................agUcaagauguuggcauagcu....................................................................   1     1    Fm3
....................aggcaagauguuggcauGgcu....................................................................   9     1    Fm3
....................aggcaagauguuggcGuagcu....................................................................   1     1    Fm3
....................Nggcaagauguuggcauagcu....................................................................   1     1    Fm3
```

**Mature** **Star**

```
ucgagacaugaacucagauuaggcaagauguuggcauagcugagucguaguuuugaaaaccucagcuguaccccauauugccaccuuuggguuuauaaauucaacaug
....................agAcaagauguuggcauagcu....................................................................   2     1   Fm3
....................aggcaagauguuggAauagcu....................................................................   4     1   Fm3
....................aggcaagauguuggcauCgcu....................................................................   11    1   Fm3
....................aggcaagauguuggcauagcu....................................................................   4136  0   Fm3
....................aggcaagauguuggcaGagcu....................................................................   15    1   Fm3
....................aggAaagauguuggcauagcu....................................................................   2     1   Fm3
....................aggcaagaCguuggcauagcu....................................................................   2     1   Fm3
....................aggcaagaugAuggcauagcu....................................................................   9     1   Fm3
....................aggcaagCuguuggcauagcu....................................................................   2     1   Fm3
....................aggcaagauguAggcauagcu....................................................................   1     1   Fm3
....................Uggcaagauguuggcauagcu....................................................................   3     1   Fm3
....................aggcaagauguAggcauagcug...................................................................   2     1   Fm3
....................aggcaaCauguuggcauagcug...................................................................   1     1   Fm3
....................aggcaagauguuggcauagcuA...................................................................   39    1   Fm3
....................Cggcaagauguuggcauagcug...................................................................   3     1   Fm3
....................aggcaagauguCggcauagcug...................................................................   2     1   Fm3
....................aggcaagauguuggcauGgcug...................................................................   21    1   Fm3
....................aggcaagauguuggUauagcug...................................................................   1     1   Fm3
....................aggcaagaugAuggcauagcug...................................................................   17    1   Fm3
....................aAgcaagauguuggcauagcug...................................................................   2     1   Fm3
....................aggcaagaCguuggcauagcug...................................................................   1     1   Fm3
....................agCcaagauguuggcauagcug...................................................................   1     1   Fm3
....................aggcaagauguuggcauUgcug...................................................................   9     1   Fm3
....................aggAaagauguuggcauagcug...................................................................   1     1   Fm3
....................aggcaagauguugUcauagcug...................................................................   1     1   Fm3
....................Nggcaagauguuggcauagcug...................................................................   6     1   Fm3
....................agAcaagauguuggcauagcug...................................................................   1     1   Fm3
....................aggcaagauAuuggcauagcug...................................................................   2     1   Fm3
....................aggcaagauguuggcaCagcug...................................................................   14    1   Fm3
....................aggcaagauguugAcauagcug...................................................................   1     1   Fm3
....................aggcaagauguuggcauagcuU...................................................................   469   1   Fm3
....................aggcaUgauguuggcauagcug...................................................................   1     1   Fm3
....................aggcaagauguuggcauCgcug...................................................................   21    1   Fm3
....................aggcaagauguugCcauagcug...................................................................   1     1   Fm3
....................aggcaagauguuggcauagcug...................................................................   6359  0   Fm3
....................aggcaagaugGuggcauagcug...................................................................   4     1   Fm3
....................aggcaagauguuggcaAagcug...................................................................   11    1   Fm3
....................aggcaagauguuggcauagGug...................................................................   2     1   Fm3
....................aggcaaUauguuggcauagcug...................................................................   1     1   Fm3
....................aggcaagauguuggcauagUug...................................................................   10    1   Fm3
....................aggcaagUuguuggcauagcug...................................................................   2     1   Fm3
....................aggcaagaugCuggcauagcug...................................................................   8     1   Fm3
....................aggcaagauguuggcaGagcug...................................................................   15    1   Fm3
....................aggcaagauguuggAauagcug...................................................................   2     1   Fm3
....................aggcaagauguuUgcauagcug...................................................................   3     1   Fm3
....................aUgcaagauguuggcauagcug...................................................................   2     1   Fm3
....................aggcaagauUuuggcauagcug...................................................................   4     1   Fm3
....................aggcaagauguuAgcauagcug...................................................................   2     1   Fm3
....................Uggcaagauguuggcauagcug...................................................................   13    1   Fm3
....................aggcaagGuguuggcauagcug...................................................................   2     1   Fm3
....................agUcaagauguuggcauagcug...................................................................   4     1   Fm3
....................aCgcaagauguuggcauagcug...................................................................   2     1   Fm3
....................aggcaagauguuggcauagcCg...................................................................   1     1   Fm3
....................aggcaagauguuggcUuagcug...................................................................   1     1   Fm3
....................aggcaagauguuggcauaAcug...................................................................   1     1   Fm3
....................aggcaCgauguuggcauagcug...................................................................   1     1   Fm3
....................aggcaagCuguuggcauagcug...................................................................   1     1   Fm3
....................aggcaagauguuggcaNagcuga..................................................................   1     1   Fm3
....................aggcaagauguuggcauagAuga..................................................................   1     1   Fm3
....................aggcaagauguuggcauUgcuga..................................................................   29    1   Fm3
....................aggcaaCauguuggcauagcuga..................................................................   4     1   Fm3
....................aggcaagauguuggcauagcugU.................................................................   2314  1   Fm3
....................aggcaagauguCggcauagcuga..................................................................   3     1   Fm3
....................Nggcaagauguuggcauagcuga..................................................................   20    1   Fm3
....................aggcaagauguuggcauCgcuga..................................................................   50    1   Fm3
....................aggcaagUuguuggcauagcuga..................................................................   2     1   Fm3
....................aggcGagauguuggcauagcuga..................................................................   4     1   Fm3
....................aggcaagauguuggcauagcugG.................................................................   2     1   Fm3
....................aggcaagauguuggcaAagcuga..................................................................   25    1   Fm3
....................agUcaagauguuggcauagcuga..................................................................   3     1   Fm3
```

**Mature** **Star**

```
ucgagacaugaacucagauuaggcaagauguuggcauagcugagucguaguuuugaaaaccucagcuguaccccauauugccaccuuuggguuuauaaauucaacaug
....................aggcaagauguuCgcauagcuga.................................................................   3      1   Fm3
....................aggcaagauguuggUauagcuga.................................................................   1      1   Fm3
....................aggcaNgauguuggcauagcuga.................................................................   1      1   Fm3
....................aggcaagaAguuggcauagcuga.................................................................   1      1   Fm3
....................aggcaagauguuUgcauagcuga.................................................................   1      1   Fm3
....................aggcaagauguuggcauaAcuga.................................................................   2      1   Fm3
....................aggcaCgauguuggcauagcuga.................................................................   1      1   Fm3
....................aggcaagauguuggcauagcuAa.................................................................   17     1   Fm3
....................aggAaagauguuggcauagcuga.................................................................   2      1   Fm3
....................aCgcaagauguuggcauagcuga.................................................................   4      1   Fm3
....................Uggcaagauguuggcauagcuga.................................................................   17     1   Fm3
....................agCcaagauguuggcauagcuga.................................................................   1      1   Fm3
....................aggcaagauguuggcauagcuUa.................................................................   10     1   Fm3
....................aggcaagauguuggcGuagcuga.................................................................   2      1   Fm3
....................aggcaagCuguuggcauagcuga.................................................................   2      1   Fm3
....................aggcCagauguuggcauagcuga.................................................................   2      1   Fm3
....................aggcaagauguuggcaCagcuga.................................................................   44     1   Fm3
....................aggGaagauguuggcauagcuga.................................................................   1      1   Fm3
....................aggcaagauguuggAauagcuga.................................................................   3      1   Fm3
....................aggcaagaugGuggcauagcuga.................................................................   17     1   Fm3
....................aggcaagauguuggcauaCcuga.................................................................   1      1   Fm3
....................aggcaagauguuggcauagcugC.................................................................   4      1   Fm3
....................aggcaagauUuuggcauagcuga.................................................................   1      1   Fm3
....................aggcaagauguuggcUuagcuga.................................................................   3      1   Fm3
....................aggcaagauguuggcaGagcuga.................................................................   51     1   Fm3
....................aUgcaagauguuggcauagcuga.................................................................   4      1   Fm3
....................aggcaagauguuggcauagcuga.................................................................   14217  0   Fm3
....................aAgcaagauguuggcauagcuga.................................................................   1      1   Fm3
....................aggUaagauguuggcauagcuga.................................................................   2      1   Fm3
....................aggcaagauAuuggcauagcuga.................................................................   2      1   Fm3
....................aggcaUgauguuggcauagcuga.................................................................   2      1   Fm3
....................aggcaagauguuAgcauagcuga.................................................................   2      1   Fm3
....................aggcaaUauguuggcauagcuga.................................................................   2      1   Fm3
....................aggcaagauguuggGauagcuga.................................................................   1      1   Fm3
....................aggcaagaugAuggcauagcuga.................................................................   32     1   Fm3
....................aggcaaNauguuggcauagcuga.................................................................   1      1   Fm3
....................aggcaagauguugUcauagcuga.................................................................   6      1   Fm3
....................aggcaGgauguuggcauagcuga.................................................................   2      1   Fm3
....................aggcaagauguugAcauagcuga.................................................................   1      1   Fm3
....................aggcaagauguuggcauGgcuga.................................................................   48     1   Fm3
....................Gggcaagauguuggcauagcuga.................................................................   4      1   Fm3
....................Cggcaagauguuggcauagcuga.................................................................   1      1   Fm3
....................aggcaagaugCuggcauagcuga.................................................................   7      1   Fm3
....................aggcaagauguuggcauaUcuga.................................................................   5      1   Fm3
....................aggcUagauguuggcauagcuga.................................................................   2      1   Fm3
....................agAcaagauguuggcauagcuga.................................................................   4      1   Fm3
....................aggcaagauguuggcauagcugaU................................................................   590    1   Fm3
....................aggcaagauguuggcauagcugaC................................................................   2      1   Fm3
....................aggcaagauguuggcauagcugag................................................................   9      0   Fm3
....................aggcaagauguuggcauagcugaA................................................................   22     1   Fm3
....................aggcaagauguuggcauagcugaAu...............................................................   146    1   Fm3
....................aggcaagauguuggcauagcugaUu...............................................................   366    1   Fm3
....................aggcaagauguuggcauagcugagu...............................................................   18     0   Fm3
.....................ggcaagauguuggcauagcu...................................................................   5      0   Fm3
.....................Ugcaagauguuggcauagcug..................................................................   1      1   Fm3
.....................ggcaagauguuggcauagcug..................................................................   13     0   Fm3
.....................ggcaagauguuggcauagcuga.................................................................   29     0   Fm3
.....................ggcaagauguuCgcauagcuga.................................................................   1      1   Fm3
.....................ggcaagauguuggcauagcugaUu...............................................................   4      1   Fm3
.....................ggcaagauguuggcauagcugaAu...............................................................   2      1   Fm3
......................gcaagauguuggcauagcug..................................................................   1      0   Fm3
.......................caagauguuggcauagcugagu...............................................................   5      0   Fm3
.............................................................ucagcuguaccccauauugcc..........................   6      0   Fm3
...............................................................agcuguaccccauauugccacc.......................   5      0   Fm3

...................uaggcaagauguuggcauagcug..................................................................   1      0   Mm3
....................aggcaagauguuggcaua......................................................................   3      0   Mm3
....................aggcaagauguuggcauU......................................................................   1      1   Mm3
....................aggcaagauguuggcauag.....................................................................   12     0   Mm3
....................aggcaagaugGuggcauag.....................................................................   1      1   Mm3
```

Mature Star

```
ucgagacaugaacucagauuaggcaagauguuggcauagcugagucguaguuuugaaaaccucagcuguaccccauauugccaccuuuggguuuauaaauucaacaug
....................aggcaagauguuggcauagU....................................................................  6    1  Mm3
....................Cggcaagauguuggcauagc....................................................................  1    1  Mm3
....................aggcaagauguuggcauagA....................................................................  1    1  Mm3
....................aggcaagauguuggcauCgc....................................................................  1    1  Mm3
....................aggcaagauguuggcauGgc....................................................................  1    1  Mm3
....................aggcaagauguuggcauagc....................................................................  65   0  Mm3
....................Nggcaagauguuggcauagcu...................................................................  1    1  Mm3
....................aggcaaNauguuggcauagcu...................................................................  1    1  Mm3
....................Uggcaagauguuggcauagcu...................................................................  1    1  Mm3
....................aggcaagauguuggcauagUu...................................................................  1    1  Mm3
....................aggcaagauguuggcauGgcu...................................................................  1    1  Mm3
....................aggcaagaugCuggcauagcu...................................................................  1    1  Mm3
....................aggcaagauguuggcauCgcu...................................................................  3    1  Mm3
....................aggcaagauguuggcauagcu...................................................................  206  0  Mm3
....................aggcaagauguuggcauagcG...................................................................  6    1  Mm3
....................aggcGagauguuggcauagcu...................................................................  2    1  Mm3
....................aggcaagauguuggcaAagcu...................................................................  1    1  Mm3
....................aggcaagauguuggcauagcuC..................................................................  1    1  Mm3
....................aggcaagaugAuggcauagcug..................................................................  1    1  Mm3
....................aggcaagauguuggcauagcGg..................................................................  5    1  Mm3
....................aggcaagauguuggcauGgcug..................................................................  1    1  Mm3
....................aggcaagauguuggcauUgcug..................................................................  4    1  Mm3
....................aggcaagauguuggcauagcuA..................................................................  5    1  Mm3
....................aggcaagauguuggcauCgcug..................................................................  1    1  Mm3
....................aggcaagauguuggcauagcug..................................................................  249  0  Mm3
....................aggcaagauguuggcauagcuU..................................................................  31   1  Mm3
....................aggcaagauguuggcaGagcug..................................................................  1    1  Mm3
....................aggcaagauguuggcauUgcuga.................................................................  1    1  Mm3
....................aggcaagauguuggcauaAcuga.................................................................  1    1  Mm3
....................aggcaagauguuggcauCgcuga.................................................................  3    1  Mm3
....................aggcaagauguuggcauGgcuga.................................................................  2    1  Mm3
....................aggcaagauguuggcaCagcuga.................................................................  2    1  Mm3
....................aggcaagauguuggcGuagcuga.................................................................  1    1  Mm3
....................aggcaagauguuggcaAagcuga.................................................................  1    1  Mm3
....................aggcaagauguuggcauagcuga.................................................................  807  0  Mm3
....................aUgcaagauguuggcauagcuga.................................................................  1    1  Mm3
....................aggcaaAauguuggcauagcuga.................................................................  1    1  Mm3
....................aggcaagauguCggcauagcuga.................................................................  2    1  Mm3
....................aCgcaagauguuggcauagcuga.................................................................  1    1  Mm3
....................aggcaagauguuggcauagcugG.................................................................  16   1  Mm3
....................Gggcaagauguuggcauagcuga.................................................................  1    1  Mm3
....................aggcaagauguuggcaGagcuga.................................................................  2    1  Mm3
....................aggcaagaGguuggcauagcuga.................................................................  1    1  Mm3
....................aggcaagauguuggcauagcuUa.................................................................  1    1  Mm3
....................Uggcaagauguuggcauagcuga.................................................................  1    1  Mm3
....................aggcaagauguuggcUuagcuga.................................................................  1    1  Mm3
....................aggcaagaugGuggcauagcuga.................................................................  1    1  Mm3
....................aggcaagauguuggcauagcuAa.................................................................  3    1  Mm3
....................aggcaagUuguuggcauagcuga.................................................................  2    1  Mm3
....................aggcaagaugAuggcauagcuga.................................................................  1    1  Mm3
....................aggcaagauguuggcauagcugU.................................................................  196  1  Mm3
....................aggcaagauguuggcauagcugaU................................................................  42   1  Mm3
....................aggcaagauguuggcauagcugaA................................................................  3    1  Mm3
....................aggcaagauguuggcauagcugaAu...............................................................  12   1  Mm3
....................aggcaagauguuggcauagcugaUu...............................................................  21   1  Mm3
....................aggcaagauguuggcauagcugagu...............................................................  2    0  Mm3
.....................ggcaagauguuggcauagcug..................................................................  1    0  Mm3
.....................ggcaagauguuggcauagcuga.................................................................  5    0  Mm3
...............................................................agcuguaccccauauugccacc.......................  1    0  Mm3
...............................................................agcuguaccccauauuCccacc.......................  1    1  Mm3
...............................................................agcuguaccccauauugccaccA......................  1    1  Mm3
```
